# Molecular Properties and Functional Divergence of the Dehydroascorbate Reductase Gene Family in Lower and Higher Plants

**DOI:** 10.1371/journal.pone.0145038

**Published:** 2015-12-18

**Authors:** Yuan-Jie Zhang, Wei Wang, Hai-Ling Yang, Yue Li, Xiang-Yang Kang, Xiao-Ru Wang, Zhi-Ling Yang

**Affiliations:** National Engineering Laboratory for Tree Breeding, College of Biological Sciences and Technology, Beijing Forestry University, Beijing, China; Institute of Genetics and Developmental Biology, Chinese Academy of Sciences, CHINA

## Abstract

Dehydroascorbate reductase (DHAR), which reduces oxidized ascorbate, is important for maintaining an appropriate ascorbate redox state in plant cells. To date, genome-wide molecular characterization of DHARs has only been conducted in bryophytes (*Physcomitrella patens*) and eudicots (e.g. *Arabidopsis thaliana*). In this study, to gain a general understanding of the molecular properties and functional divergence of the DHARs in land plants, we further conducted a comprehensive analysis of DHARs from the lycophyte *Selaginella moellendorffii*, gymnosperm *Picea abies* and monocot *Zea mays*. DHARs were present as a small gene family in all of the land plants we examined, with gene numbers ranging from two to four. All the plants contained cytosolic and chloroplastic DHARs, indicating dehydroascorbate (DHA) can be directly reduced in the cytoplasm and chloroplast by DHARs in all the plants. A novel vacuolar DHAR was found in *Z*. *mays*, indicating DHA may also be reduced in the vacuole by DHARs in *Z*. *mays*. The DHARs within each species showed extensive functional divergence in their gene structures, subcellular localizations, and enzymatic characteristics. This study provides new insights into the molecular characteristics and functional divergence of DHARs in land plants.

## Introduction

As a major antioxidant, ascorbic acid (Asc) provides crucial protection against oxidative damage in plants [1]. Besides its antioxidant role, Asc functions as a cofactor for a large number of key enzymes, and a regulator of cell division and growth in plants [2,3]. Once used, Asc is oxidized to monodehydroascorbate, a short-lived radical, which is reduced to Asc by monodehydroascorbate reductase (MDHAR) or disproportionates to Asc and dehydroascorbate (DHA) [4]. Dehydroascorbate reductase (DHAR) catalyzes the reduction of DHA to Asc using glutathione (GSH) as a reducing substrate [2].

DHAR plays important roles in regulating the cellular Asc redox state. For example, overexpression of DHAR in tobacco leaves resulted in a more reduced Asc redox state, while suppression of DHAR had the opposite effect [5]. Much evidence has shown that DHAR is crucial for protecting plants against oxidative injury. A tropical fig lacking heat-stable DHAR activity in its leaves exhibited high light sensitivity, indicating a role for DHAR in protecting plants against photoinhibition [6]. An *Arabidopsis* mutant lacking cytosolic DHAR activity had lower apoplastic Asc content and was highly ozone sensitive [7]. Overexpression of a rice cytosolic DHAR gene conferred enhanced salt stress tolerance to rice plants by maintaining the Asc pool, ion homeostasis and redox homeostasis [8]. Transgenic tobacco overexpressing the *Arabidopsis* cytosolic DHAR gene showed enhanced tolerance to ozone and drought stresses [9].

DHARs usually exist as small gene families in plants. For example, the rice genome contains two DHARs, and the *Populus trichocarpa*, *S*. *moellendorffii* and *P*. *patens* genomes contain three DHARs each [10]. Of the three *P*. *patens* DHARs, *PpDHAR1* was expressed under all growth conditions tested, *PpDHAR2* was selectively expressed in response to specific treatments, and *PpDHAR3* expression was not detected by PCR in any of the samples tested [10]. Although the three *Populus tomentosa* DHARs were expressed in all tissues examined, they showed different subcellular localizations. PtoDHAR1 was localized to the chloroplast, while PtoDHAR2 and PtoDHAR3 showed typical cytosolic localization [11]. Three of the four *Arabidopsis* DHARs were examined for their catalytic activities, which differed towards the DHA substrate [12]. These results show that the DHAR members in plants might have functionally diverged.

Previous genome-wide analyses of the DHAR gene family integrating sequence analysis, gene expression, protein subcellular localization and biochemical characterization have been conducted on the bryophyte *P*. *patens* and eudicots such as *Arabidopsis* [10,12]. However, the molecular characteristics and functional divergence of DHAR families in other land plants have not been investigated. In this study, we conducted a comprehensive analysis of the gene sequences, gene structures, gene expression patterns, subcellular localization and biochemical characteristics of the DHARs in the lycophyte *S*. *moellendorffii*, gymnosperm *P*. *abies* and monocot *Z*. *mays*. By systematic analysis of the DHAR gene families in different land plants, we have gained a general understanding of the functional divergence of DHARs in land plants.

## Results

### Sequence characteristics of the DHAR genes in land plants

To investigate the sequence characteristics of DHARs in land plants, we conducted a joint sequence analysis of DHAR genes from a bryophyte (*P*. *patens*), a lycophyte (*S*. *moellendorffii*), gymnosperms (*P*. *abies* and *Pinus taeda*), and angiosperm eudicots (*A*. *thaliana* and *P*. *trichocarpa*) and monocots (*Z*. *mays* and *Brachypodium distachyon*). *P*. *abies*, *P*. *taeda* and *B*. *distachyon* each contained two DHAR gene copies. *P*. *patens*, *S*. *moellendorffii* and *P*. *trichocarpa* each contained three copies. Four DHAR genes existed in the *Z*. *mays* and *A*. *thaliana* genomes, respectively. The DHARs from *S*. *moellendorffii*, *P*. *abies*, and *Z*. *mays* were cloned from the cDNAs of the above three species. Although the predicted *SmDHAR3* gene encodes protein with complete DHAR domain, no DNA fragments amplified from *S*. *moellendorffii* cDNA for *SmDHAR3* can be translated into protein containing complete DHAR domain. So *SmDHAR3* was considered to be a pseudogene. The predicted *SmDHAR3* splice variant encoding protein with complete DHAR domain was used for subsequent sequence analysis.

**Fig 1:**
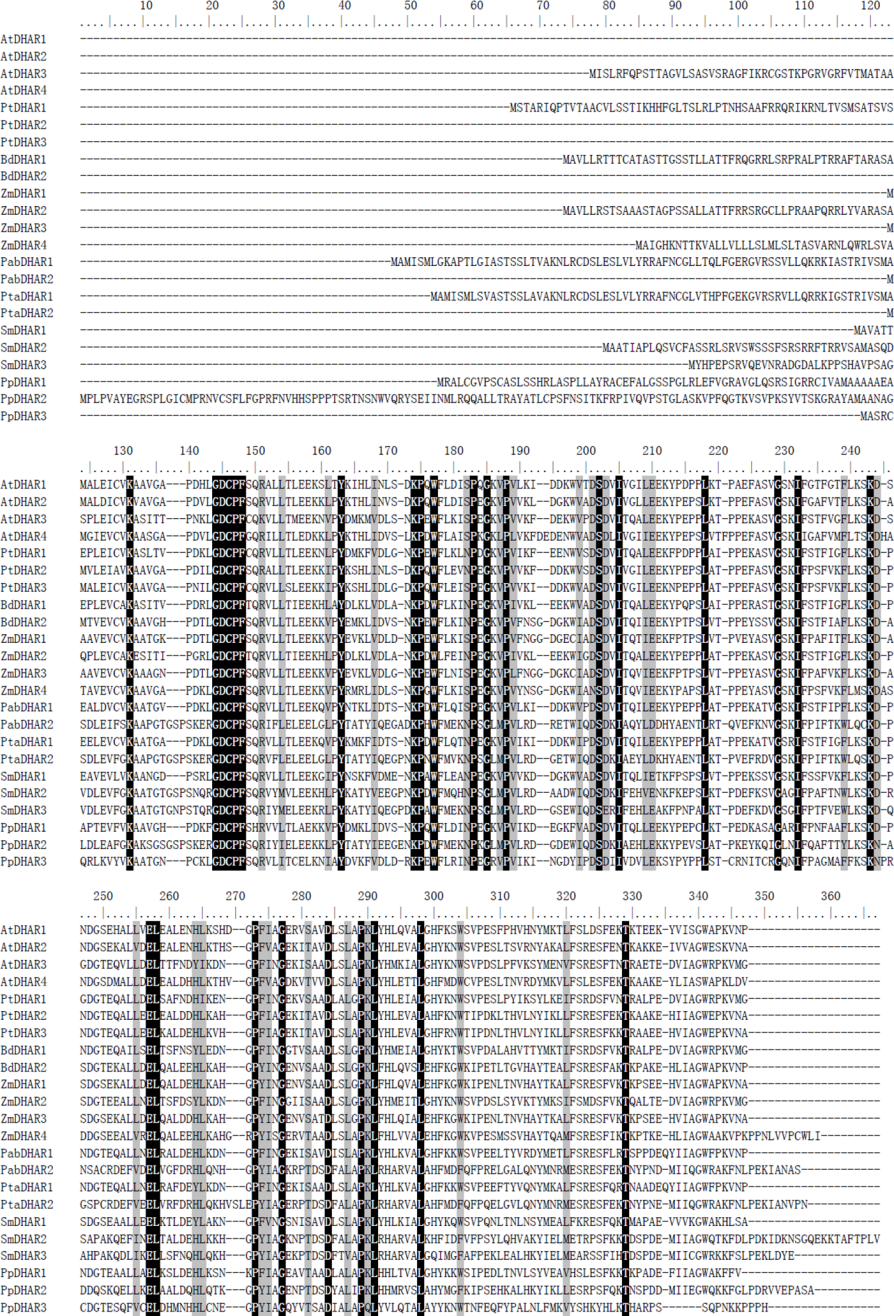
Multiple sequence alignment of plant dehydroascorbate reductases (DHARs). Conserved residues in all plant DHARs are marked in black. At, *A*. *thaliana*; Pt, *P*. *trichocarpa*; Bd, *B*. *distachyon*; Zm, *Z*. *mays*; Pab, *P*. *abies*; Pta, *P*. *taeda*; Sm, *S*. *moellendorffii*; Pp, *P*. *patens*.

The land plant DHAR genes examined encoded proteins of different sizes, ranging from 212 to 349 amino acids. Each species contained DHARs that were more than 25 residues longer than one of the others within the species. Multiple protein sequence alignment showed that the protein length differences were mainly due to excess peptides at the N-terminus (Fig 1). These excess peptides were predicted to be putative signal peptides that targeted the DHARs to specific subcellular locations. After removing the highly variable peptides, we conducted pairwise comparison of the DHAR domain sequences in the above eight species. The pairwise sequence identity of DHARs within each species was > 32%, and all land plant DHARs showed > 29% pairwise sequence identity in their DHAR domains.

**Fig 2:**
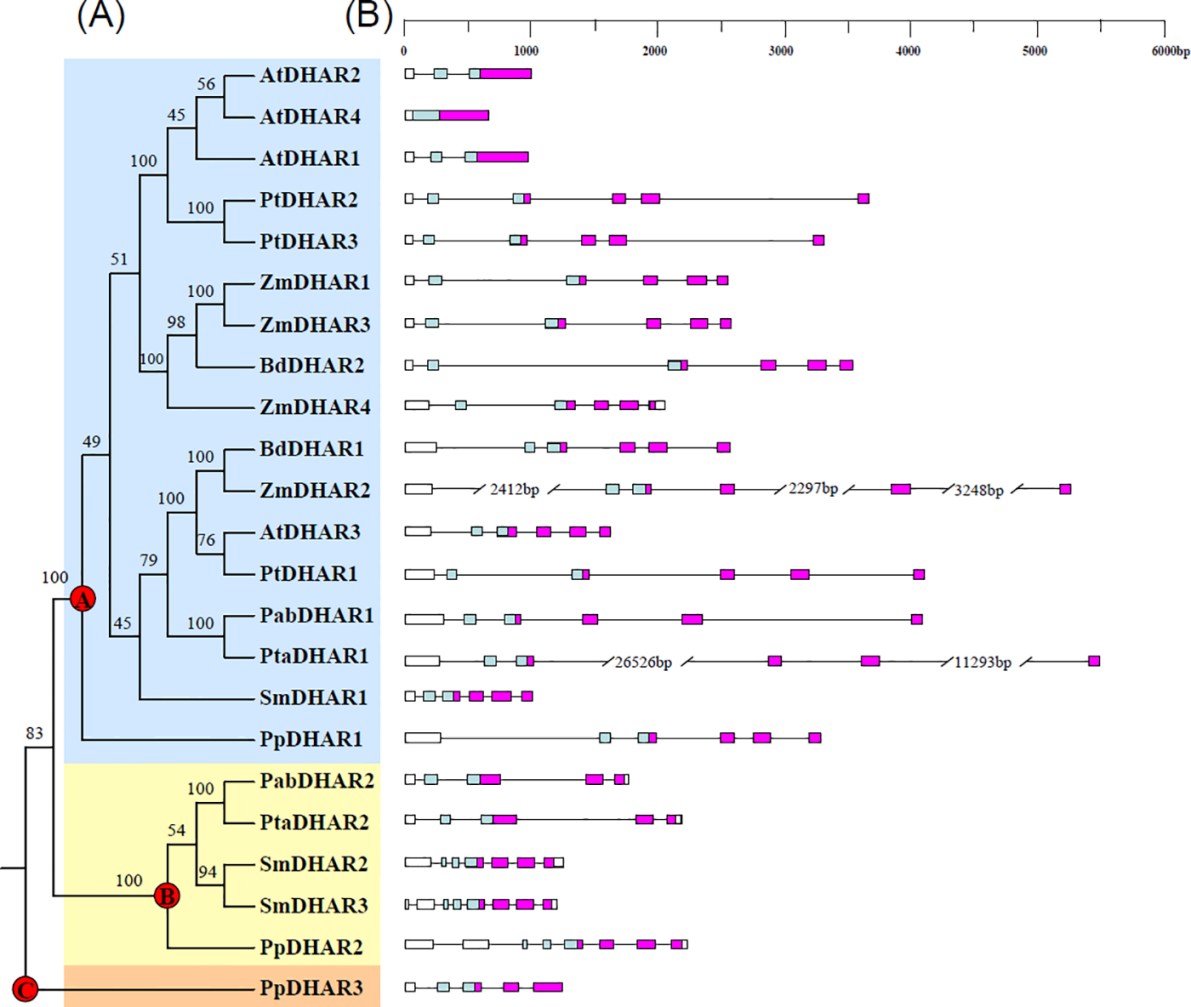
Phylogenetic relationships among land plant dehydroascorbate reductases (DHARs) (A), and gene structures (B). Numbers on branches indicate the bootstrap values calculated from 100 replicates. Clades I, II and III DHARs are shaded blue, yellow and brown, respectively. The three ancestral genes of land plant DHARs are indicated by red circles. In (B), the GST N-terminal domain and C-terminal domain are highlighted by the blue and purple boxes, respectively, while introns are indicated as lines.

Phylogenetic relationships among the land plant DHARs were reconstructed using a neighbor-joining procedure. The 23 DHARs from the above eight species formed three clades in the phylogenetic tree with 100% bootstrap values. The three clades were termed clade I, II and III DHARs. Clade I contained DHARs from all eight species assessed in this study. Clade II contained DHARs from a bryophyte (*P*. *patens*), a lycophyte (*S*. *moellendorffii*), and gymnosperms (*P*. *abies* and *P*. *taeda*), but not from angiosperms. Clade III only contained one member from *P*. *patens* (Fig 2A). A previous study postulated that three ancestral DHAR copies might have existed in the common ancestor of land plants [10]. In this study, based on phylogenetic analysis, we also identified three ancestral DHAR copies (A, B and C, corresponding to clades I, II and III, respectively) in the common ancestor of land plants (Fig 2A). The ancestral DHAR A has been retained in all of the land plants, and expanded in angiosperms. The ancestral DHAR B has been lost in angiosperms, while the ancestral DHAR C has only been retained in bryophytes.

Except three *Arabidopsis* DHARs (*AtDHAR1*, *2* and *4*), the DHARs in clade I all had five introns, suggesting that the ancestral DHAR A might have five introns. The three-exon/two-intron structure of *AtDHAR1* and *AtDHAR2* might result from intron loss events, and the one-exon structure of *AtDHAR4* might result from a retrotransposition event. Highly variable gene structures were observed in clade II. The bryophyte DHAR gene *PpDHAR2* and the lycophyte DHAR gene *SmDHAR3* each had seven introns. *SmDHAR2* had a seven-exon/six-intron gene structure. Both *PabDHAR2* and *PtaDHAR2* had five-exon/four-intron gene structures. *PpDHAR3* in clade III had four introns (Fig 2B).

### Expression of DHAR genes from *S*. *moellendorffii*, *P*. *abies* and *Z*. *mays*

**Fig 3:**
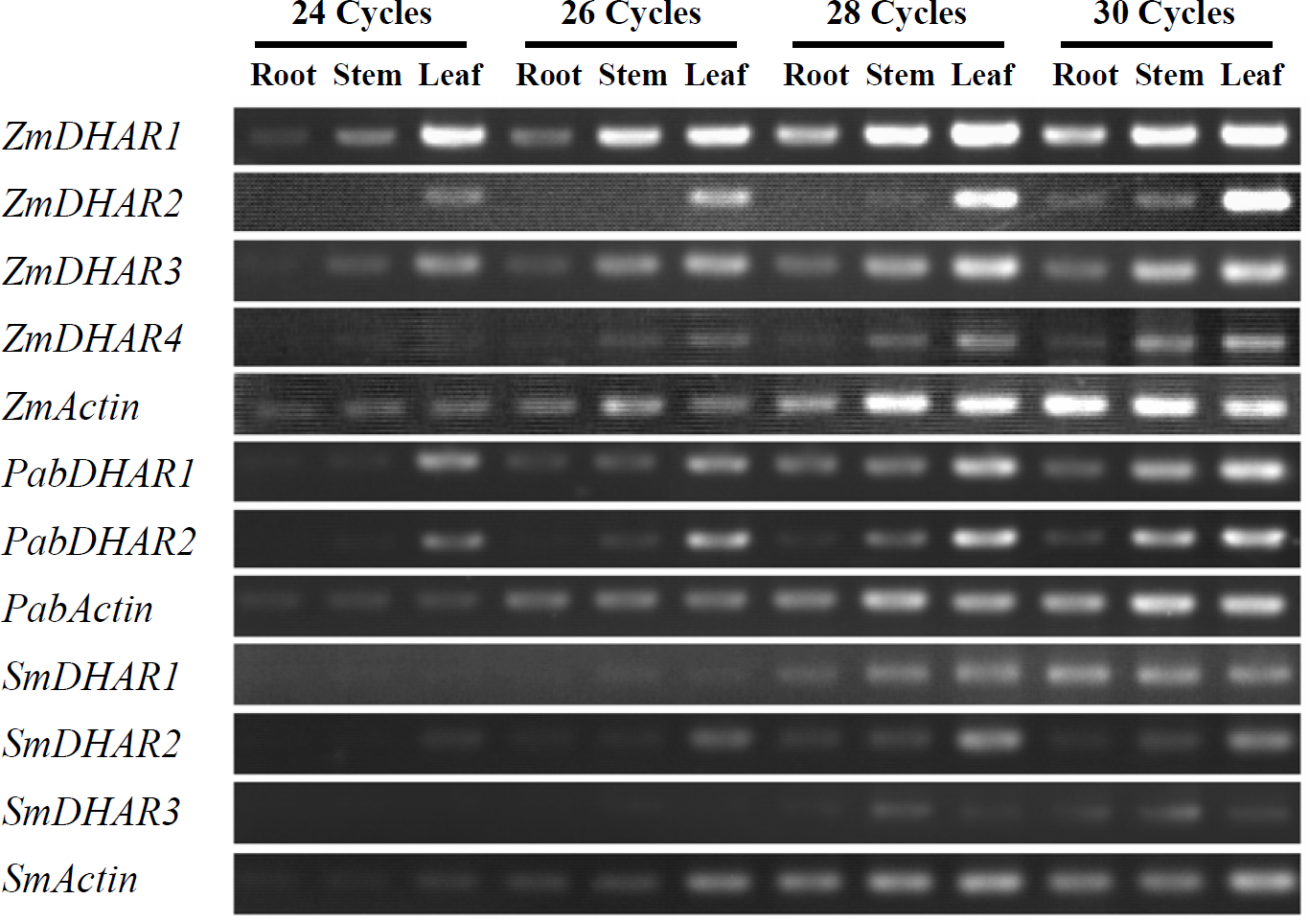
Expression of the DHAR genes in various tissues of *Z*. *mays*, *P*. *abies*, and *S*. *moellendorffii*.

The expression patterns of the DHAR genes from *S*. *moellendorffii*, *P*. *abies*, and *Z*. *mays* in three tissues including roots, stems and leaves were investigated. Some DHAR genes (e.g. *ZmDHAR1* and *PabDHAR1*) were detected in all of the tested tissues by different numbers of amplification cycles (24, 26, 28 or 30), while *ZmDHAR2* was only detected in all of the tested tissues by 30 amplification cycles. All the DHAR genes from the above three species were expressed in all of the tissues examined by 30 PCR amplification cycles (Fig 3). The expression level of some DHARs was different in different tissues. For example, at 24 cycles, *ZmDHAR1* and *ZmDHAR2* showed much higher expression level in leaf than in root and stem. The two DHARs of *P*. *abies* also showed the highest expression in leaf. At 28 cycles, the expression level of *SmDHAR3* was higher in stem than in other two tissues.

### Subcellular localization of DHARs from *S*. *moellendorffii*, *P*. *abies* and *Z*. *mays*

**Fig 4:**
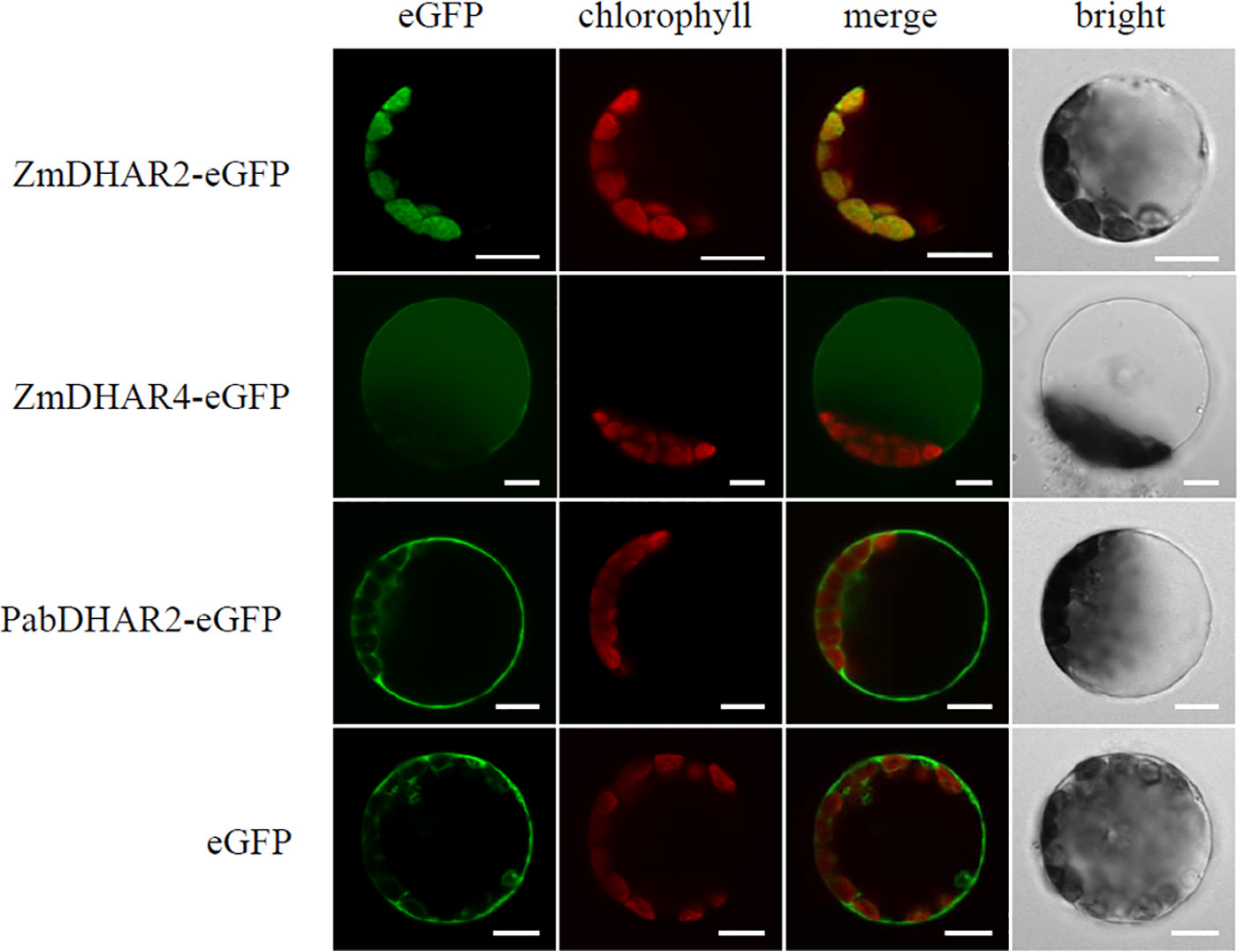
Subcellular localizations of DHAR proteins in *Arabidopsis* protoplasts. eGFP signal (green) and chlorophyll autofluorescence (red) were observed using confocal laser scanning microscopy, and an overlay is shown in yellow. Scale bars are 10 μm.

We first predicted the subcellular localization of the DHARs from *S*. *moellendorffii*, *P*. *abies* and *Z*. *mays* using the TargetP 1.1 server (http://www.cbs.dtu.dk/services/TargetP/). The location assignment is based on the predicted presence of any N-terminal presequences: the chloroplast transit peptide (cTP), mitochondrial targeting peptide (mTP) or secretory pathway signal peptide (SP). Among the eight DHARs from the above three species, one DHAR in each species (SmDHAR2, PabDHAR1 and ZmDHAR2) was predicted to contain a cTP, ZmDHAR4 was predicted to contain a SP, and the other four (SmDHAR1, PabDHAR2, ZmDHAR1 and ZmDHAR3) were predicted not to contain any of the above three signal peptides. To experimentally verify the prediction results, translational fusions of the DHARs to the N-terminus of the enhanced green fluorescent protein (eGFP) were generated and transiently expressed in *Arabidopsis* leaf protoplasts. Visualization by confocal microscopy showed that the GFP signals of three DHAR-eGFP fusions (SmDHAR2-, PabDHAR1- and ZmDHAR2-eGFP) were co-localized with the red autofluorescence signals of chlorophyll, demonstrating that they were localized to the chloroplast. In cells expressing the ZmDHAR4-eGFP fusion, the fluorescence was seen in the vacuole, indicating ZmDHAR4 was localized to the vacuole. The fluorescence patterns of other four DHAR-eGFP fusions (SmDHAR1, PabDHAR2, ZmDHAR1 and ZmDHAR3) were similar to that of eGFP alone, where the fluorescence was found throughout the cytosol (Fig 4).

### Expression and purification of DHAR proteins from *S*. *moellendorffii*, *P*. *abies* and *Z*. *mays*

**Fig 5:**
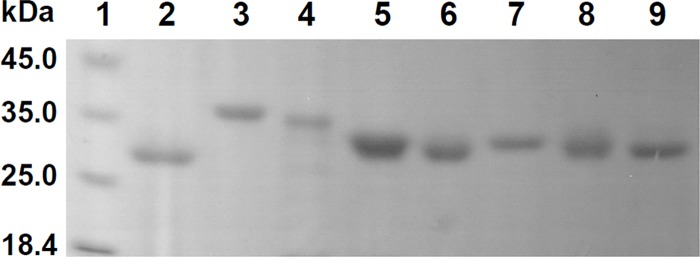
SDS-PAGE of purified DHAR proteins. Lane 1, molecular mass markers with the sizes shown on the left in kDa; lanes 2–9, SmDHAR1, SmDHAR2, PabDHAR1, PabDHAR2, ZmDHAR1, ZmDHAR2, ZmDHAR3 and ZmDHAR4, respectively.

SmDHAR2, PabDHAR1, ZmDHAR2 and ZmDHAR4 all contained a signal peptide at their N-terminus. After removing the signal peptides, the eight DHARs from *S*. *moellendorffii*, *P*. *abies* and *Z*. *mays* were over-expressed in *E*. *coli*. Except PabDHAR1, all of the DHARs were expressed mainly as soluble proteins in *E*. *coli* at 37°C. After the induction temperature was lowered to 20°C, PabDHAR1 was also expressed as a soluble protein. The eight DHARs were purified using a Ni Sepharose High Performance column. In SDS-PAGE analysis, all of the purified recombinant proteins showed a single band (Fig 5).

### Enzymatic activity and steady-state kinetics of the DHAR proteins from *S*. *moellendorffii*, *P*. *abies* and *Z*. *mays*

The biochemical characteristics of the DHAR proteins from *S*. *moellendorffii*, *P*. *abies* and *Z*. *mays* were assayed using GSH and DHA as substrates. Among the eight DHARs, SmDHAR1 showed the highest catalytic activity (26.15 ± 0.94 μmol min^−1^ mg^−1^), while PabDHAR2 showed the lowest catalytic activity (0.18 ± 0.00 μmol min^−1^ mg^−1^). The highest catalytic activity was 118.86-, 6.44- and 5.94-fold higher than the lowest catalytic activity in *S*. *moellendorffii*, *P*. *abies* and *Z*. *mays*, respectively (Table 1).

**Table 1:** Comparison of the specific activities and kinetic constants of land plant dehydroascorbate reductases (DHARs) towards the substrates dehydroascorbate (DHA) and glutathione (GSH). Pto, *P*. *tomentosa*.

	Activities	Kinetic constants		Subcellular localization	
DHARs	(μmol/min per mg)	*K*_m_^GSH^ (mM)	*K*_m_^DHA^ (mM)		References
**ZmDHAR1**	18.45±0.32	0.740±0.044	0.068±0.010	cytosol	The present study
**ZmDHAR2**	13.47±0.19	2.507±0.159	0.068±0.009	chloroplast	The present study
**ZmDHAR3**	19.12±0.26	1.079±0.053	0.150±0.004	cytosol	The present study
**ZmDHAR4**	3.22±0.16	2.883±0.129	0.119±0.007	vacuole	The present study
**PabDHAR1**	1.16±0.06	2.705±0.055	0.113±0.001	chloroplast	The present study
**PabDHAR2**	0.18±0.00	1.344±0.034	0.118±0.001	cytosol	The present study
**SmDHAR1**	26.15±0.94	1.466±0.025	0.115±0.004	cytosol	The present study
**SmDHAR2**	0.22±0.01	2.255±0.139	0.248±0.015	chloroplast	The present study
**PpDHAR1**	54.1±0.87	3.64±0.34	0.17±0.01	cytosol and chloroplast	(Liu et al. 2012)
**PpDHAR2**	1.03±0.05	1.08±0.10	0.13±0.02	chloroplast	(Liu et al. 2012)
**PtoDHAR1**	53.02	3.75±0.77	0.07±0.01	chloroplast	(Tang et al. 2013)
**PtoDHAR2**	49.89	2.28±0.22	0.23±0.02	cytosol	(Tang et al. 2013)
**PtoDHAR3**	38.32	2.47±0.39	0.48±0.02	cytosol	(Tang et al. 2013)

The apparent kinetic constants of the DHAR proteins were assayed with various concentrations of GSH and DHA. The DHARs in the above three species showed apparent *K*_m_ values for GSH ranging from 0.74 to 2.88 mM, and for DHA ranging from 0.07 to 0.25 mM. ZmDHAR1 showed the highest affinity (lowest *K*_m_^GSH^ and *K*_m_^DHA^) towards both the GSH and DHA substrates (Table 1).

### Temperature and pH profiles of the DHARs from *S*. *moellendorffii*, *P*. *abies* and *Z*. *mays*

**Fig 6:**
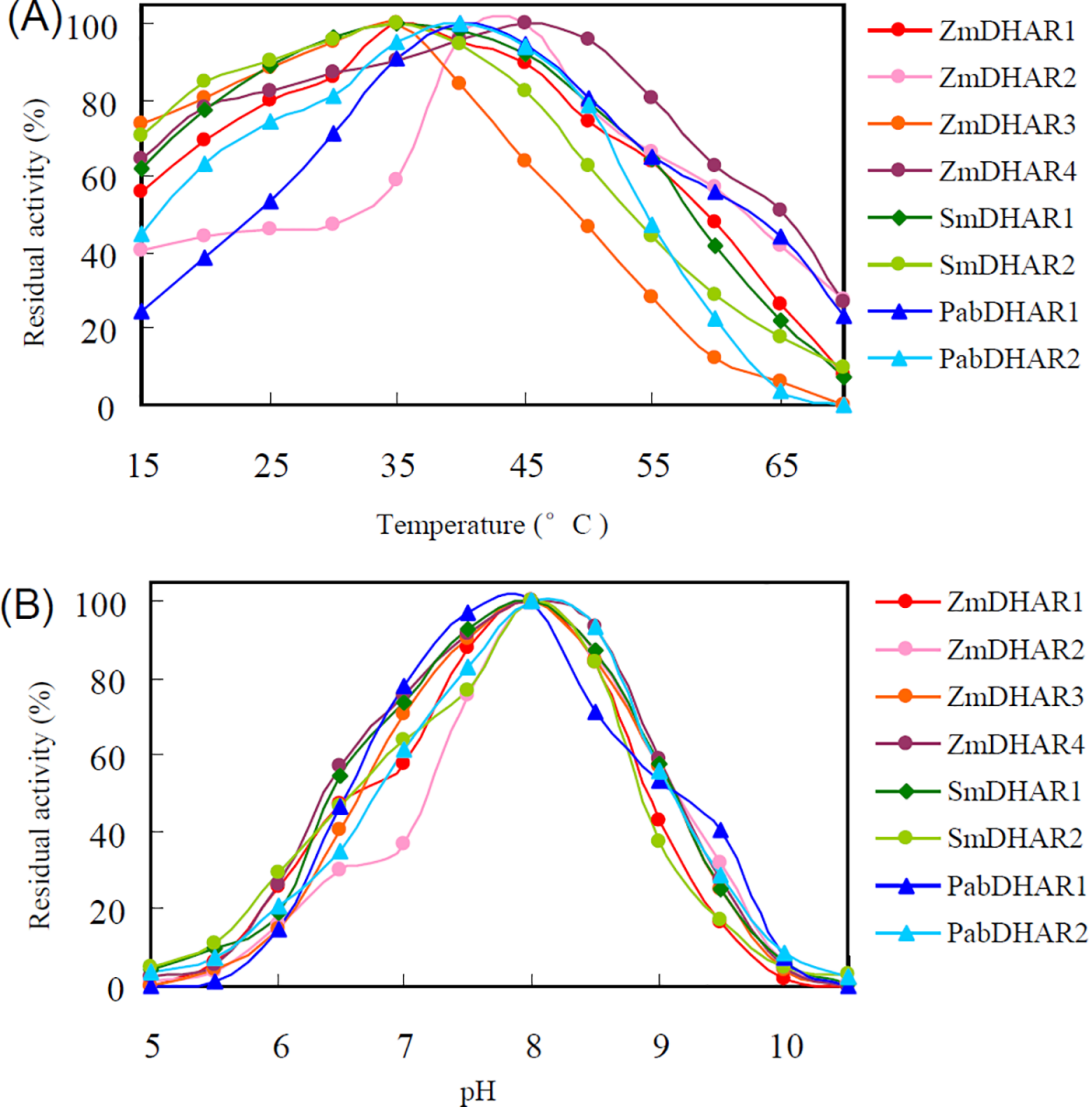
Effects of temperature (A) and pH (B) on DHAR activity.

The eight DHARs from *S*. *moellendorffii*, *P*. *abies* and *Z*. *mays* showed broad temperature optimums for enzymatic activity. All of the eight DHARs retained more than 45% of their maximum enzymatic activity between 25 and 50°C. Optimum catalytic temperature variation was also observed among the DHARs. At 60°C, SmDHAR2, PabDHAR2 and ZmDHAR3 retained no more than 29% of their maximum enzymatic activity, while the other DHARs retained more than 41% of their maximum enzymatic activity. At 25°C, PabDHAR1 and ZmDHAR2 retained no more than 54% of their maximum enzymatic activity, while all of the other DHARs retained more than 75% of their maximum enzymatic activity (Fig 6A).

A pH profile analysis showed that all eight DHARs of the three species had maximum enzymatic activities at pH 8.0. They almost completely lost their enzymatic activity below pH 5.5 or above pH 10.0. ZmDHAR3, ZmDHAR4, SmDHAR1, PabDHAR1 and PabDHAR2 retained more than 53% of their maximum enzymatic activity between pH 7.0 and 9.0, while ZmDHAR1 and SmDHAR2 retained more than 57% of their maximum enzymatic activity between pH 7.0 and 8.5. ZmDHAR2 showed no more than 37% of its maximum enzymatic activity below pH 7.0 (Fig 6B).

## Discussion

Dehydroascorbate reductase (DHAR), which reduces oxidized ascorbate, is important for maintaining an appropriate ascorbate redox state in plant cells. Two to four full-length DHAR copies have been found in each land plant species examined so far. A joint phylogenetic analysis of land plant DHARs showed that three ancestral DHAR copies (A, B and C) might have existed in the common ancestor of land plants, which is consistent with the previous study [10]. The ancestral DHAR gene C has only been retained in bryophytes and the ancestral DHAR B has been lost in angiosperms, while the ancestral DHAR A has been retained in all of the land plants and expanded in angiosperms. Protein catalytic activity analysis showed that the catalytic activities of all the DHARs examined in clade II were lower than those of clade III, indicating that the ancestral DHAR copy with higher catalytic activity had a higher possibility to be retained and expand throughout its evolutionary history.

Ascorbate is mainly present in the cytosol and chloroplast [13]. After use, ascorbate is oxidized to MDHA and then disproportionated to DHA [4]. DHAR can reduce DHA to ascorbate [2]. The correct localization of DHAR proteins is essential for their function [14]. Both cytosolic and chloroplastic DHARs were identified in plants, such as the bryophyte *P*. *patens*, and eudicots *A*. *thaliana* and *P*. *tomentosa* [10,11,12]. In this study, we found that cytosolic and chloroplastic DHARs also existed in the lycophyte *S*. *moellendorffii*, gymnosperm *P*. *abies* and monocot *Z*. *mays*, indicating that all land plants might contain cytosolic and chloroplastic DHARs. Thus, DHA generated in the cytosol and chloroplast can be directly reduced to ascorbate by corresponding DHARs in land plants.

Although the DHARs in each species shared high sequence identities in their DHAR domains, clear differences were observed in their gene structures, subcellular localizations, and enzymatic characteristics, which suggested functional divergence. For example, SmDHAR1, PabDHAR2, and ZmDHAR1/3 were localized to the cytoplasm, while SmDHAR2, PabDHAR1 and ZmDHAR2 were localized to the chloroplast. SmDHAR1 and PabDHAR1 had 118.86- and 6.44-fold higher catalytic activity to DHA than SmDHAR2 and PabDHAR2, respectively. Previous studies have shown that the patterns of functional divergence of DHARs differ in *P*. *tomentosa* and *A*. *thaliana* [11]. The chloroplastic DHAR had the highest catalytic activity in *P*. *tomentosa*, while the chloroplast-localized AtDHAR3 showed much lower catalytic activity than the cytosolic AtDHAR1 [11,12]. In this study, we found that the chloroplastic PabDHAR1 had much higher catalytic activity than the cytosolic PabDHAR2, whereas the catalytic activities of chloroplastic DHARs were lower than those of cytosolic DHARs in *S*. *moellendorffii* and *Z*. *mays*. Our research supports that the patterns of functional divergence of DHARs are different in different plant species.

Ascorbate appears to be synthesized in cytosol, and then translocated into the chloroplast, vacuole and apoplast following the concentration gradient [15]. As DHAR exists in cytosol and chloroplast, the ascorbate can be regenerated from DHA by DHAR in cytosol and chloroplast. No apoplastic and vacuolar DHARs were identified in previous studies. It has been postulated that DHA formed in the vacuole and apoplast may be transported to the cytoplasm to be reduced and then translocated back into the vacuole and apoplast [16]. In this study, a DHAR (ZmDHAR4) with dehydroascorbate reductase activity was found to localize to the vacuole in *Z*. *mays*. So the DHA generated in vacuoles in *Z*. *mays* may not need to be transported to cytosol for re-reduction, but be directly reduced in vacuole in *Z*. *mays*. We did not identify any vacuolar DHAR in other species investigated in this study, indicating the reduction of DHA by DHAR in vacuole may only exist in *Z*. *mays*.

Of all the DHARs examined, only ZmDHAR4 was localized to vacuole. The vacuolar localization of ZmDHAR4 may be the result of subcellular relocalization. Signal peptide prediction showed that ZmDHAR4 contained a secretory pathway signal peptide (SP) which targets the ZmDHAR4 to vacuole. Except ZmDHAR4, none of the DHARs contained SP. The specific vacuolar localization of ZmDHAR4 may be the result of the gain of a vacuolar signal peptide. It has been proposed that protein subcellular relocalization is one source of retention and functional diversification of duplicate genes. Changes in the subcellular location of eukaryotic duplicate proteins can positively modify their function and therefore be beneficial to the organism [17]. It has been shown that ascorbate may play important roles in detoxifying the hydroperoxide generated in or diffused into vacuoles [16]. Direct reduction of DHA in the vacuole catalyzed by vacuolar DHAR avoids the transport of DHA to the cytoplasm and the transport of reduced ascorbate back to the vacuole, enabling faster reuse of ascorbate to detoxify reactive oxygen species in the vacuole. Thus, the vacuolar subcellular relocalization of *Z*. *mays* DHAR may confer *Z*. *mays* specific ability against oxidative stress.

## Materials and Methods

### DHAR gene identification

We conducted TBLASTN searches of the genome databases of *P*. *patens*, *S*. *moellendorffii*, *P*. *abies*, *P*. *taeda*, *A*. *thaliana*, *P*. *trichocarpa*, *Z*. *mays* and *B*. *distachyon* using an *Arabidopsis* DHAR protein sequence (GenBank accession number NP_173387) as the template with default algorithm parameters. Of the two putative DHAR genes identified from the *P*. *abies* genome, one was truncated at its N-terminus. As this truncated DHAR is located at a scaffold terminus in the *P*. *abies* genome database, the truncation may be due to the incomplete sequencing of the *P*. *abies* genome. To get its full-length sequence, we searched the *Picea glauca* EST database using the truncated gene as a template. Based on the identified EST sequences that were most similar to the truncated *P*. *abies* DHAR gene, a primer pair was designed to amplify the full-length gene from *P*. *abies* complementary DNA (cDNA). The amplified DNA fragment was then cloned into the pEASY-T3 vector (TransGen, Beijing, China), and sequenced in both directions to verify the gene sequence. Based on the full-length cDNA sequence of the amplified DHAR gene, another primer pair was designed to amplify the genomic sequence from *P*. *abies* DNA. The amplified DNA fragment was also cloned into the pEASY-T3 vector, and sequenced in both directions to verify the sequence. The identified putative DHAR candidates were then analyzed using the National Center for Biotechnology Information (NCBI) conserved domain search to confirm the presence of the DHAR domain in their protein structures. For the subsequent subcellular localization and enzymatic activity analysis, the DHARs of *S*. *moellendorffii*, *P*. *abies* and *Z*. *mays* were amplified from cDNAs of the above three species, respectively, cloned into pEASY-T3 vector and sequenced in both directions.

### Phylogenetic analyses

Full-length DHAR protein sequences were aligned with the MUSCLE software (http://www.ebi.ac.uk/Tools/msa/muscle/) and manually adjusted using BioEdit [18]. The DHAR domain protein sequences were used to reconstruct phylogenetic relationships using a Neighbor-Joining (NJ) procedure in the MEGA software with the p-distance amino acid substitution model [19]. One hundred bootstrap replicates were performed in each analysis.

### Detection of DHAR gene transcripts in various tissues

To investigate the expression patterns of the DHAR genes of *S*. *moellendorffii*, *P*. *abies* and *Z*. *mays* in various tissues, total RNAs were isolated from roots, stems and leaves of the three species. The isolated RNAs were then treated with RNase-free DNase and reverse-transcribed into cDNA using an RNA PCR Kit (AMV) version 3.0 (TaKaRa, Dalian, China). The *Actin* genes of the three species were used as internal controls. PCR was performed in a volume of 25 μL containing 3 μL first-strand cDNA, 2.5 μL TaKaRa 10× PCR buffer, 0.125 μL TaKaRa Ex Taq (5 U/μL), 2 μL dNTP (2.5 mM each), and 0.4 pmol primer. PCR conditions consisted of an initial denaturation of 3 min at 94°C, followed by cycles of 30 s at 94°C, 40 s at 60°C and 1 min at 72°C with a final extension of 3 min at 72°C. The number of cycles used for amplification with each primer pair was 24, 26, 28 or 30. The PCR products were each analyzed using a 1% agarose gel and were validated by DNA sequencing.

### Subcellular localization of DHAR proteins

To investigate the subcellular localization of the DHAR proteins, the DHAR genes were subcloned into the pPSN transgenic vector to generate C-terminal green fluorescent protein (GFP) fusions driven by the 35S promoter [11]. Colonies containing potential appropriate recombinant plasmids were confirmed by sequencing. The pPSN vectors containing the DHAR genes were transiently transformed into protoplasts of *Arabidopsis thaliana* by polyethylene glycol—calcium transformation [20]. The transformed protoplasts were then observed with a confocal laser microscope from 1 to 3 days after the transformation. GFP fluorescence was excited with a 488 nm laser, and chlorophyll autofluorescence was excited using a 543 nm laser.

### DHAR protein expression and purification

To investigate the enzymatic activities of the DHAR proteins, the DHAR genes were subcloned into the pET-30a (Novagen, Madison, WI, USA) vector to express N-terminal 6×His-tagged fusion proteins. Recombinant plasmids containing the DHAR genes were then transformed into *Escherichia coli* BL21 (DE3). Overnight cultures of the *E*. *coli* BL21 harboring the recombinant plasmids were diluted 1:100, and grown until the optical density (*A*_600_) reached 0.5. Synthesis of the recombinant DHAR proteins was induced by isopropyl-beta-d-thiogalactopyranoside (IPTG) at a final concentration of 0.1 mM. After incubation at 37 or 20°C overnight, the bacteria were harvested by centrifugation at 8000 × g for 3 min at 4°C, resuspended in binding buffer (20 mM sodium phosphate, 0.5 M NaCl, and 20 mM imidazole, pH 7.4), and disrupted by cold sonication. The soluble fraction was separated from the insoluble fraction by centrifugation at 10,000 × g for 10 min at 4°C. The supernatant (soluble fraction) was then mixed with Ni Sepharose High Performance affinity media (Amersham Pharmacia Biotech, Piscataway, NJ, USA) that had been preequilibrated with binding buffer for 40 min at 25°C. After the unbound contaminants were removed by washing with binding buffer, the bound proteins were eluted with elution buffer (20 mM sodium phosphate, 0.5 M NaCl, and 500 mM imidazole, pH 7.4).

### Enzymatic activity characterization

The dehydroascorbate reductase activity was determined by measuring the increase in *A*_265_ due to the formation of ascorbate (ε = 14.0 mM^−1^ cm^−1^). The reaction mixture contained 90 mM potassium phosphate buffer, pH 6.5, 0.42 mM GSH, 0.17 mM DHA, and various amounts of proteins. For a blank, the elution buffer was used instead of the purified DHAR proteins. All assays were carried out at 25°C. Sodium dodecyl sulfate-polyacrylamide gel electrophoresis (SDS-PAGE) was conducted on a 10% separating gel and a 5% stacking gel. Protein concentrations were determined by measuring absorbance at 280 nm.

The apparent kinetic constants of the DHAR proteins were studied in assays with various concentrations of GSH and DHA. The apparent *K*_m_ values for GSH were measured using GSH concentrations ranging from 0.125 to 2.0 mM in the presence of 0.17 mM DHA. The apparent *K*_m_ values for DHA were measured using DHA concentrations ranging from 0.013 to 1.677 mM in the presence of 0.42 mM GSH. The kinetic parameters were derived from nonlinear regression analysis by the Hyper32 program available at http://homepage.ntlworld.com/john.easterby/hyper32.html. The dependence of DHAR activity on temperature was examined in 90 mM potassium phosphate buffer (pH 6.5) at various temperatures from 15°C to 70°C at 5°C intervals. The dependence of DHAR activity on pH was measured according to the method described by Yuen and Ho [21].
